# Fault Diagnosis Method for Pumping Station Units Based on the tSSA-Informer Model

**DOI:** 10.3390/s25206458

**Published:** 2025-10-18

**Authors:** Qingqing Tian, Hongyu Yang, Yu Tian, Lei Guo

**Affiliations:** 1 School of Water Conservancy, North China University of Water Resources and Electric Power, Zhengzhou 450046, China; tq10078@126.com (Q.T.); 15554436569@163.com (H.Y.); 2 China Institute of Water Resources and Hydropower Research, Beijing 100048, China; ty10078@126.com; 3 Henan Water Conservancy Investment Group Co., Ltd., Zhengzhou 450002, China; 4 Henan Water Valley Innovation Technology Research Institute Co., Ltd., Zhengzhou 450000, China

**Keywords:** fault diagnosis, t-distribution strategy Sparrow Search Algorithm, Informer Model, vibration signal processing, anti-noise performance

## Abstract

**Highlights:**

**What are the main findings?**

- The proposed tSSA-Informer model achieves 90.85% diagnostic accuracy under strong noise (SNR = −9 dB) and 61.32% accuracy with only 10% labeled samples, outperforming SSA-Informer, GA-Informer, and other comparative models.
- The tSSA algorithm optimizes Informer hyperparameters 7.74% faster than the original SSA, and the integrated data preprocessing (IQR + CWT + KPCA) effectively enhances the representation of non-stationary vibration features.

**What is the implication of the main finding?**

- The model’s anti-noise and small-sample adaptability precisely addresses the “multi-interference, scarce samples” dilemma of pumping station unit fault diagnosis in practical engineering.
- The tSSA-optimized Informer framework provides a referable paradigm for hyperparameter tuning and time-series feature mining in mechanical fault diagnosis fields.

**Abstract:**

To address the problems of noise sensitivity, insufficient modeling of long-term time-series dependence, and high cost of labeled data in the fault diagnosis of pumping station units, an intelligent diagnosis method integrating the improved Sparrow Search Algorithm (tSSA) and Informer model is proposed in this study. Firstly, an adaptive t-distribution strategy is introduced into the Sparrow Search Algorithm to dynamically adjust the degree of freedom parameters of the mutation operator, balance global search and local development capabilities, avoid the algorithm converging to the origin, and enhance optimization accuracy, with time complexity consistent with the original SSA. Secondly, by combining the sparse self-attention and self-attention distillation mechanisms of Informer, the model’s ability to extract key features of long sequences is optimized, and its hyperparameters are adaptively optimized via tSSA. Experiments were conducted based on 12 types of fault vibration data acquired from pumping station units. Outliers were removed using the interquartile range (IQR) method, and dimensionality reduction was achieved through kernel principal component analysis (KPCA). The results indicate that the average diagnostic accuracy of tSSA-Informer under noise-free conditions reaches 98.73%, which is significantly higher than that of models such as SSA-Informer and GA-Informer; under noise interference of SNR = −1 dB, it still maintains an accuracy of 87.47%, outperforming comparative methods like 1D-DCTN; when the labeled sample size is reduced to 10%, its accuracy is 61.32%, which is more than 40% higher than that of traditional models. These results verify the robustness and practicality of the proposed method in strong-noise and small-sample scenarios. This study provides an efficient solution for the intelligent fault diagnosis of complex industrial equipment.

## 1. Introduction

The vertical pump unit is the core equipment of the pumping station. As a typical complex rotating machine, the operational stability of the pumping station directly determines the overall safety and operational efficiency of the pumping station, which is crucial for the reliable implementation of key water conservancy tasks such as farmland irrigation and urban water supply [1]. However, the economic loss caused by undetected faults of the unit is also significant. The expected maintenance cost for a single fault of a pumping station in a small and medium-sized water conservancy project is approximately CNY 23,000. The estimated annual maintenance cost is approximately CNY 270,000. The annual economic loss corresponding to large-scale water conservancy project pumping stations is approximately CNY 1.8 million [2,3]. Research shows that abnormal vibration is the core characteristic signal reflecting mechanical faults of such equipment. However, the vibration signals of pump units are often affected by fluctuations in working conditions, showing non-stationary and non-linear characteristics. They are also prone to superimposing environmental and equipment background noise, resulting in high signal complexity [4,5]. Therefore, how to efficiently separate noise from complex vibration signals and accurately extract fault features has become a core issue that urgently needs to be addressed in the field of fault diagnosis for vertical pump units.

Current research on fault diagnosis mainly covers three links: signal denoising, feature extraction, and feature recognition. Traditional methods have obvious limitations: Fourier transform-based methods are restricted by fixed window functions and thus struggle to resist high-frequency noise [6]; empirical mode decomposition-based methods are susceptible to end effects and mode mixing, with poor performance under strong noise interference [7]. Although deep learning-based data-driven methods (e.g., RNN, LSTM) have shown potential in processing time-series data, RNN, due to its simple structure, is prone to gradient explosion when handling long time-series vibration signals [8,9], while LSTM has limited capability in modeling non-stationary features [10,11]. As Demassey et al. [12] found, under the high-dimensional temporal characteristics of daily pumping station scheduling, the deep model overfits on the training set, and the verification error significantly increases. Regularization or a hybrid model is needed to suppress overfitting. The Transformer model has effectively addressed the issue of long-term time-series dependence modeling through the self-attention mechanism and has achieved high accuracy in bearing fault diagnosis [13]. For instance, Bao et al. converted vibration signals into time-frequency maps and input them into the Transformer, achieving an accuracy of 98.45% [14]. Wang et al. pointed out in their review that the average accuracy of the Transformer on the CWRU dataset exceeds 99% [15]. However, the self-attention mechanism of the Transformer essentially relies on pairwise correlation calculations between all global time points, leading to a quadratic increase in computational complexity with sequence length, which makes it difficult for industrial deployment. Additionally, it has limited capability in modeling instantaneous impact features in bearing faults [16], and its characteristic of equally focusing on all time points causes fault features to be easily submerged under strong noise.

The Informer model proposed by Zhang et al. [17] has significantly reduced the computational complexity of the Transformer through the Probsparse self-attention and self-attention distillation mechanisms, making it particularly adept at handling long time-series prediction tasks. It has been widely applied in fields such as medical care, energy, and transportation, yet research on it in the field of fault diagnosis remains insufficient [18,19,20]. Given that both vibration signals and audio signals have temporal correlation, the Informer has significant potential in rolling bearing fault diagnosis. However, the model’s performance highly depends on hyperparameter settings, so many scholars optimize the Informer’s parameters via optimization algorithms. For instance, Rao et al. proposed combining the Informer with GP-BO, reducing the lithium battery voltage prediction error to the 9 mV level and achieving convergence 30% faster than grid search [21]. Schneider et al. proposed a multi-objective grey wolf optimizer (MOGWO) to balance the Informer’s diagnostic accuracy, inference speed, and memory usage [22]. Liu et al. developed a SHAP-XGBoost analyzer to quantify the contribution of various parameters in the Informer to accuracy and visualize the decision path [23]. Nie et al. employed TPE to optimize the Informer’s hyperparameters, increasing the average accuracy to 96.52% compared with traditional RNN/LSTM models [24]. Although existing optimization methods have significantly improved model performance, these algorithms themselves have problems such as slow convergence and susceptibility to falling into local optima.

To address the aforementioned limitations, this study proposes a fault diagnosis method for pumping station units that integrates the improved Sparrow Search Algorithm (tSSA) and Informer, based on 12 types of fault vibration data collected from a modular rotor test bench. Specifically, an adaptive t-distribution strategy is introduced to dynamically adjust the degree of freedom parameters of the SSA mutation operator, balancing global search and local exploitation capabilities and breaking through the origin convergence limitation. Meanwhile, by combining the sparse self-attention mechanism and self-attention distillation mechanism of Informer, the method reduces the computational complexity of long sequences and enhances the ability to extract noise-resistant features. Aiming to improve diagnostic accuracy and anti-interference capability under complex working conditions, this study provides a theoretical basis and technical support for fault diagnosis of pumping station units in complex scenarios such as high noise and variable rotational speed.

## 2. Research Methods

### 2.1. Improved Sparrow Search Algorithm

#### 2.1.1. Adaptive T-Distribution Strategy Improves the Sparrow Search Algorithm

In 2020, Xue et al. [25] drew inspiration from the behavioral characteristics of sparrows foraging for food and evading predators, and they developed the Sparrow Search Algorithm (SSA). Compared with other swarm intelligence optimization algorithms, SSA has the characteristics of strong optimization capability, fast convergence, high stability, and strong robustness [26]. In this algorithm, the behavior of sparrows foraging for food can be regarded as a process of searching for the optimal solution within a specific spatial range, and the goal of sparrow search is to find the global optimal value in this process. In SSA, if a discoverer has a good fitness value, it will obtain high-quality food more quickly. The foraging range of discoverers is wider than that of followers, as they need to forage for food and then provide directions for followers to find food.

The iteration formula of the discoverer during each iteration is as follows:

(1) $$X_{i,j}^{t+1}=\left\{\begin{array}{l} X_{i,j}^{t}\cdot \exp(\frac{-i}{\alpha \cdot T_{\max}}),R_{2}<S_{T}\\ X_{i,j}^{t}+Q\cdot L,R_{2}\geq S_{T} \end{array}\right.$$

where: *t* is the current iteration number; $T_{\max}$ is the maximum number of iterations; $X_{i,j}^{t}$ is the position occupied by the *i*-th sparrow in the *j*-th dimension; α is a random number, with α∈(0,1]; *R*_2_ and *S_T_* are the warning value and safety value, respectively, where $R_{2}\in [0,1]$ and $S_{T}\in [0.5,1]$; Q is a random number following a normal distribution; *L* is a 1 *× d* matrix where all elements are 1.

The position of the member is updated as follows:

(2) $$X_{i,j}^{t+1}=\left\{\begin{array}{l} Q\cdot \exp(\frac{X_{Worst}^{t}-X_{i,j}^{t}}{i^{2}}),i<\frac{2}{n}\\ X_{p}^{t+1}+\left|X_{i,j}^{t+1}-X_{P}^{t+1}\right|\cdot A^{+}\cdot L,else \end{array}\right.$$

where $X_{p}^{t+1}$ is the optimal position owned by the discoverer; $X_{Worst}^{t}$ is the global worst position; *n* is the number of sparrows in the population; A is a matrix where elements are assigned 1 or −1, with $A^{+}=A^{T}{\left(AA^{T}\right)}^{-1}$.

In the sparrow population, some sparrows can detect the occurrence of danger, which are called sentinels. Initially, the positions of sentinels in the population are randomly distributed, and their positions are updated according to the following formula:

(3) $$X_{i,j}^{t+1}=\left\{\begin{array}{l} X_{best}^{t}\cdot \beta \left|X_{i,j}^{t}-X_{best}^{t}\right|,f_{i}>f_{g}\\ X_{i,j}^{t}+K\left(\frac{X_{i,j}^{t}-X_{Worst}^{t}}{(f_{i}-f_{W})+\varepsilon }\right),f_{i}=f \end{array}\right.$$

In the formula, *X_best_* represents the global optimal position, which refers to the optimal combination of hyperparameters corresponding to the positions of all sparrow individuals during the iteration process. Its calculation logic is as follows: After initializing the population, in each generation of iteration, the fitness of all sparrow individuals is calculated first, then the position of the individual with the best fitness in the current generation is dynamically updated to *X_best_* through generation-by-generation selection; *β* and *K* are the step size control parameters. *β* is a random number that follows a normal distribution, and *K* is a random number with K∈[−1,1]; $f_{i}$ is the fitness value of the *i*-th sparrow; ε is a minimum constant to avoid the denominator becoming 0; *f_g_* is the fitness value corresponding to *X_best_*; *f_W_* is the fitness value corresponding to the global worst position *X_Worst_*.

*X_best_* is the global optimal position; *β* and *K* are step control parameters, where *β* is a random number following a normal distribution and *K* is a random number with K∈[−1,1]; $f_{i}$ is the fitness value of the *i*-th sparrow; ε is a minimum constant to avoid the denominator becoming zero.

In Reference [25], the performance of the Sparrow Search Algorithm (SSA) was tested, and the analysis revealed that when *R*_2_ > *S_T_*, discoverers will randomly move near their current positions following a normal distribution (i.e., their values converge to the optimal position); when *R*_2_ < *S_T_*, each dimension of the sparrow’s position becomes smaller (i.e., its values converge to 0), which is not a favorable strategy. Therefore, SSA outperforms other swarm intelligence optimization algorithms by a large margin when solving functions where the optimal solution is close to the origin; conversely, its performance degrades slightly. Since the position of the optimal solution cannot be confirmed in the actual global optimization process, this study adopts the method shown in Equation (4) to eliminate the convergence toward the origin, thereby addressing the issue of low optimization accuracy of SSA when the optimal solution is far from the origin and further enhancing the algorithm’s overall global optimization capability. The revised formula for updating the discoverers’ positions is as follows:

(4) $$X_{i,j}^{t+1}=\left\{\begin{array}{l} X_{i,j}^{t}\cdot (1+Q),R_{2}>S_{T}\\ X_{i,j}^{t}\cdot Q\cdot L,R_{2}\geq S_{T} \end{array}\right.$$

Existing studies have proven that the Gaussian Distribution (GD) can enhance the search capability of individuals near the optimal point and accelerate the convergence speed of the algorithm [27]. The Cauchy Distribution (CD) can improve the exploration capability of individuals in the entire solution space and increase population diversity. Both can effectively enhance the optimization capability of the algorithm, while the t-distribution has both advantages of GD and CD. Thus, this study adopts an adaptive t-distribution strategy to improve the Sparrow Search Algorithm (SSA). Meanwhile, the impact of a small number of outliers on the t-distribution is far smaller than that on the Gaussian distribution. Building a regression model based on a long-tailed probability distribution enables a more robust model. A random number r and a probability are set: if r is less than the dynamic probability threshold, a mutation operator with the number of iterations as the degree of freedom parameter of the t-distribution is used to perturb the optimal position of the sparrow. This allows the algorithm to have good global exploitation capability in the early stage of iteration and excellent local exploration capability in the later stage. The specific process is as follows:

First, define the iteration progress factor η to quantify the optimization process; Furthermore, a function ν(t) with dynamic changes in degrees of freedom as the iteration progresses is constructed. This function enhances the global search ability in the early stage of the iteration (simulating the Cauchy distribution characteristics) and strengthens the local exploration accuracy in the later stage of the iteration (approaching the Gaussian distribution characteristics).

(5) $$\eta =\frac{t}{T_{\max}}$$

(6) ν(t)=10⋅(1−η)+2

In the formula, t represents the current number of iterations; $T_{\max}$ represents the maximum number of iterations.

Based on this, the mutation operator is expressed as follows:

(7) $$t_{D}(T)=r_{t}\cdot (x_{\mathrm{pbest},j}(t)-x_{i,j}(t))$$

Here, $r_{t}\sim \mathrm{Cauchy}(0,1)$ is a random number that follows a t-distribution (the degrees of freedom are dynamically determined by Equation (6)); $t_{D}(T)$ is a mutation operator.

Ultimately, the sparrow position update formula can be fully expanded as follows:

(8) $$X_{i,j}^{t+1}=X_{i,j}^{t}+X_{i,j}^{t}\cdot t_{D}(T)$$

Through this linkage mechanism, the mutation operator adaptively adjusts the search characteristics as the optimization progresses, effectively avoiding the decline in particle swarm diversity or local optimum problems.

#### 2.1.2. Analysis of the Time Complexity of the Improved Sparrow Search Algorithm

Time complexity analysis typically relies on three rules: population initialization, calculation of the fitness function, and update of the solution. Suppose the population size is N, and O(N) is the computational complexity of the population initialization process. Then, the computational complexity of the solution update process is O(T × N) + O(T × N × D), where T is the total number of iterations and D is the spatial dimension. Therefore, the total time complexity of the original SSA is O(N × (T × D + 1)). From the complexity of the original SSA, it can be known that tSSA only adds the adaptive T-distribution strategy, and its computational complexity is O(T × N). Therefore, the total time complexity of tSSA is O(N × (T × (D + 1) + 1)). From the above analysis, it can be seen that after introducing the adaptive T-distribution strategy into the original sparrow search algorithm, no additional computational order is added, and its time complexity is of the same order of magnitude as that of the original SSA.

### 2.2. The Principle of Informer Model

**Figure 1:**
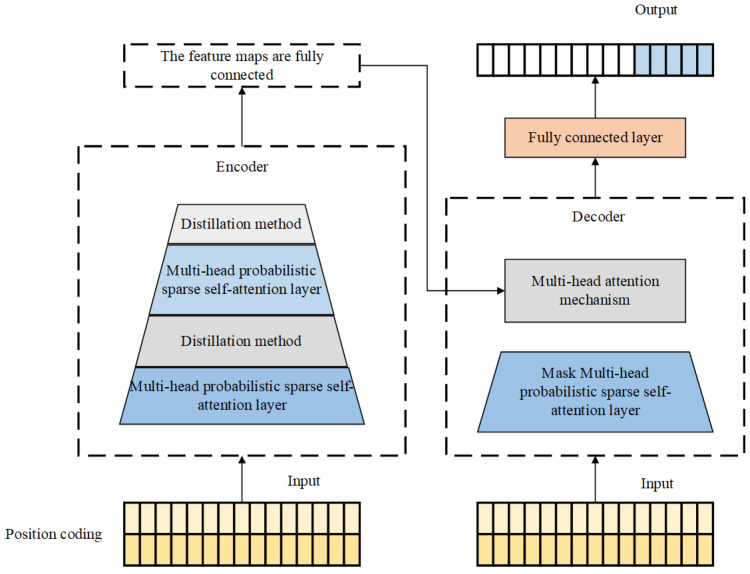
Schematic diagram of the Informer structure.

Informer is a multi-time-series prediction model composed of multiple modules, including an encoder, masked multi-head self-attention, convolutional layer, fully connected layer, Prob-sparse self-attention mechanism, and adaptive self-attention distillation mechanism [28]. Its basic architecture is shown in Figure 1.

**Figure 2:**
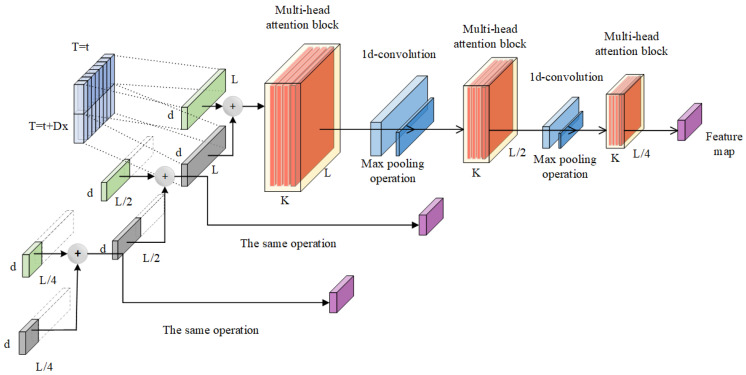
Informer encoder structure (the “the same operation” marked in the figure refers to the clearly marked “Multi-head attention block → 1d-convolution → Max pooling operation → Multi-head attention block → Feature map” on the left. A complete process with exactly the same feature processing operation).

Each encoder layer consists of a multi-head self-attention mechanism and a feed-forward network. The multi-head self-attention mechanism can capture the dependency relationships between different positions in the input sequence, extract feature representations for each position, and adopt a masking method to prevent information leakage. The encoder is composed of multiple encoder layers, as shown in Figure 2.

In the masked multi-head self-attention mechanism, each attention head uses a mask to prevent the model from using future information to predict the current value. This mechanism helps the model learn long-term dependencies in the sequence. Meanwhile, the model employs multiple attention heads to learn the dependencies between different positions in the sequence and extract features from various perspectives.

The sparse self-attention mechanism introduces a sparsity constraint based on the traditional self-attention mechanism, making attention weights more sparse. This addresses the issue of high computational complexity in traditional self-attention, enabling the model to better process long-sequence data, reduce redundant information in the input sequence, and improve the model’s generalization ability and interpretability.

The measurement formula of the traditional self-attention mechanism is as follows:

(9) $$Attention(Q,K,V)=Softmax(QK^{T}/\sqrt{d})V$$

In the formula, *Q* represents the query matrix, *K* represents the key matrix, *V* represents the value matrix, *Softmax*(*x*) represents the excitation function for normalization processing, and d represents the input dimension.

The attention metric formula for calculating the *i*-th row query matrix is as follows:

(10) $$Attention(q_{i},K,V)=\sum_{j}\frac{k(q_{i},k_{j})}{\sum_{j}k(q_{i},k_{j})}v_{j}{=}_{p(q_{i},k_{j})}[V_{j}]$$

In the formula, *q_i_*, *k_i_*, and *v_i_* represent the *i*-th row of matrices *Q*, *K*, and *V*, respectively.

However, according to the above formula, the traditional self-attention mechanism assumes that the values at each time step (or moment) have the same importance, and it usually adopts a uniform distribution to calculate the attention weights. However, in actual situations, there are often multiple time steps that do not conform to this assumption—especially for the vibration timing signal of the pumping station unit at a sampling frequency of 1000 Hz (a single sample contains 1000 time steps, and the key fault features are concentrated in local fragments such as the instantaneous impact of rotor friction and the periodic peak of misalignment faults). Uniform weights will lead to the dilution of fault characteristics by redundant timing information. Therefore, in order to more accurately assess the significance of each time step, this paper adopts the ProbSparse probabilistic sparse model to implement sparse self-attention. The core is to introduce the Kullback–Leibler (K-L) divergence as a measurement tool to achieve differentiated evaluation of each time step: the difference between the distribution of a single query vector (Q) and the key vector (K) is quantified through K-L divergence. The larger the divergence value, the more significant the deviation of the corresponding time series fragment from the random distribution, and the higher the probability of containing critical fault information. By removing the constant term and improving the calculation accuracy, the i-th sparsity measurement formula is obtained as follows:

(11) $$\bar{M}(q_{i},K)=\underset{j}{\max}\{\frac{q_{i}k_{j}^{T}}{\sqrt{d}}\}-\frac{1}{L_{K}}\sum_{j=1}^{L_{K}}\frac{q_{i}k_{j}^{T}}{\sqrt{d}}$$

In the formula, $\bar{M}(q_{i},K)$ represents the value of the maximum arithmetic mean sparse average self-attention at time $q_{i}$. The essence of this formula is to provide a quantitative basis for subsequent sparse screening by quantifying the distribution differences of “query-key”. When the value is larger, the significance of the fault features of the corresponding time series fragment is stronger, and its attention weight should be retained more.

Substituting Equation (8) into Equation (6) yields the sparse self-attention formula, as follows:

(12) $$Attention(Q,K,V)=Softmax(\bar{Q}K^{T}/\sqrt{d})V$$

The sparsity of this formula is implemented through “Top-k attention allocation”. Based on the K-L divergence sorting results calculated by Equation (10), only the Top 50 high-divergence query vectors (corresponding to a sparse density of 5%, that is, k/sequence length = 50/1000) and all key vectors are selected for attention weight calculation, and the attention weights of the remaining query vectors are directly set to 0. The determination of this sparsity level strictly conforms to the characteristics of the vibration signal of the pumping station: on the one hand, the fault-critical features in the vibration of the pumping station (such as collision impact points, misalignment periodic peaks) account for approximately 5% of the entire time series. The Top-50 screening can fully cover the core feature segments, avoiding feature omissions caused by too small k values (such as k = 30, sparse density 3%). On the other hand, by calculating the weights for only 5% of the queries, the computational complexity of the traditional self-attention O(n^2^) (where n is the sequence length) can be reduced to O(nlogn), meeting the processing requirements of long sequences with 1000 time steps while avoiding excessive k values (such as k = 80). Redundant computing and noise interference are caused by a sparse density of 8%.

By calculating the K-L divergence, several features were identified to have significant differences from the uniform distribution. Specifically, the K-L divergence values of these features are relatively large, reflecting their relatively high importance. Therefore, these feature data are screened out for further calculation, thereby effectively reducing the complexity of the calculation. The self-attention distillation mechanism can learn the relationships between different positions and the interactions between different attention layers, enabling the model to better adapt to input sequences of different lengths, improving the model’s prediction performance, and enhancing its feature representation and anti-interference capabilities.

The self-attention distillation mechanism can learn the relationships between different positions and the interactions between different attention layers, enabling the model to better adapt to input sequences of different lengths, improving the model’s prediction performance, and enhancing its feature representation and anti-interference capabilities.

The formula for the self-attention distillation mechanism is as follows:

(13) $$X_{j+1}^{t}=MaxPool(ELU(Conv1d({\left[X_{j}^{t}\right]}_{AB})))$$

where $X_{j}^{t}$ denotes the X sample at time *t* of the *j*-th layer; [.]*_AB_* denotes the self-attention block; Conv1d denotes the 1D convolution operation; MaxPool denotes the max-pooling operation; ELU denotes the activation function used.

### 2.3. tSSA-Informer Model

**Figure 3:**
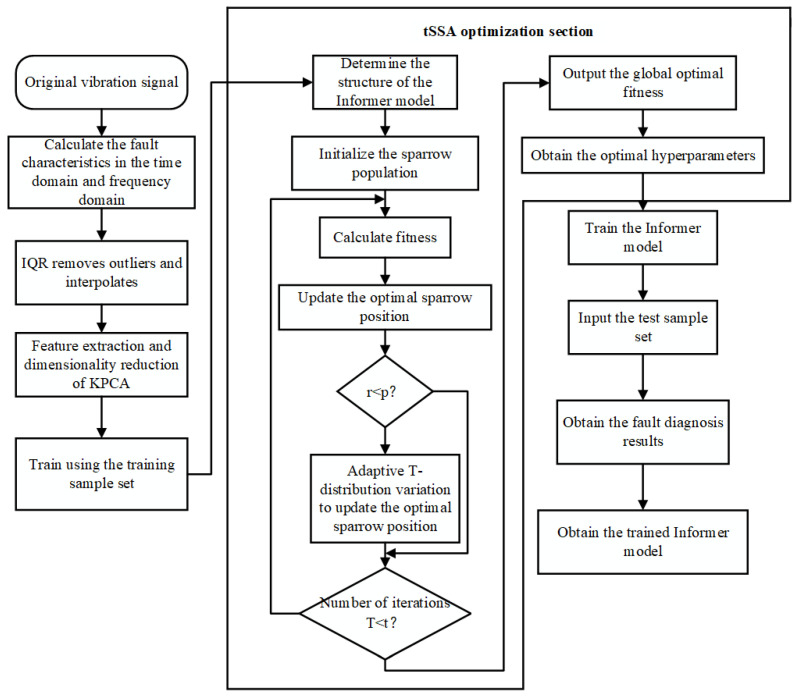
tSSA-Informer fault diagnosis process.

The diagnostic process of the tSSA-informer model is shown in Figure 3, and the specific process is as follows:

(1) Data processing: First, clarify the input and output of the tSSA-Informer fault diagnosis model. The feature parameters extracted from the vibration signals of pumping station units serve as the model input, and the fault categories of pumping station units are used as the model output. When inputting data, it is first necessary to identify and remove outliers from the data using the interquartile range (IQR) method, then correct the data via spline interpolation. The KPCA method is applied for feature extraction and dimensionality reduction, and finally, the input format of the data is adjusted to a 3D tensor suitable for the Informer model. Ultimately, the experimental data are divided into a 70% training set and a 30% test set.
(2) Set the corresponding parameters of the tSSA algorithm: population size N, proportion of discoverers P*_D_*, proportion of sentinels S*_D_*, warning value R_2_, and total number of iterations *T*; initialize the sparrow population using the initialization function; construct the Informer fault diagnosis model and determine the range of hyperparameters to be optimized.
(3) Optimize Informer hyperparameters using the tSSA algorithm: Input the experimental samples into the fault diagnosis model for classification training, take the error rate during training as the fitness value in the optimization process, and obtain the optimal Informer hyperparameters after *T* iterations.
(4) Perform fault diagnosis using the optimized model: The sample labels correspond to the fault types of pumping station units. The adaptability and effectiveness of the model are evaluated by comparing the model’s predicted output results with the true labels.

## 3. Fault Diagnosis of the Pump Station Unit Dataset

### 3.1. Experimental Configuration

All experiments are based on a unified hardware environment: The training end adopts Intel Xeon Gold 6338 CPU (32 cores), NVIDIA Tesla T4 GPU (16 GB video memory), 128 GB DDR4 memory, and the software framework is PyTorch 1.13.1 (accelerated by CUDA 11.7). The inference end simulates the edge deployment scenario of the pumping station, selecting an Intel Core i5-12400F CPU (6 cores), 16 GB of memory, and a Huawei Atlas 200 AI acceleration module (4TOPS computing power), which is in line with the configuration of industrial-grade edge devices.

### 3.2. Data Collection and Data Processing

#### 3.2.1. Data Collection

**Figure 4:**
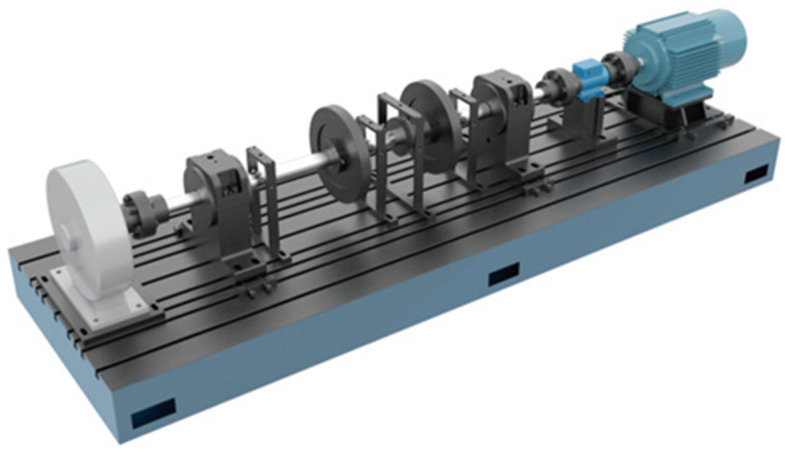
Rotor test bench.

To verify the effectiveness of the proposed method in the fault diagnosis of pumping station units, this study conducts experimental research on three typical fault modes: rotor misalignment fault; rotor-stator friction fault; misalignment and friction coupling fault. Considering that in the actual operation of the pumping station, the vibration signal will inevitably be superimposed with the electromagnetic noise of the motor, the turbulence and pressure pulsation noise of the fluid, and the random vibration noise of mechanical components such as bearings, in the data acquisition stage of this experiment, the background noise of the laboratory environment was not deliberately shielded to retain the signal characteristics close to the site. Meanwhile, subsequently, the sudden outliers caused by noise will be eliminated through the interquartile range method (IQR) in Section 3.2.2, and the high-frequency redundant noise will be suppressed in combination with the time-frequency domain filtering capability of the continuous wavelet Transform (CWT) to achieve the effective separation of fault characteristics and noise. The experiment adopted a modular rotor test bench, as shown in Figure 4. By installing a 1.0 mm precision gasket on the lower end bearing housing, the rotor parallel misalignment fault was simulated. Meanwhile, a brass contact piece was set 0.2 mm above the guide bearing to simulate the rubbing fault. During the experiment, the rotational speed adjustment from 1000 to 3000 r/min was achieved through a variable frequency control system. A total of 12 different states were constructed in this experiment. Among them, 400 sets of data (a total of 4800 samples) were collected for each bearing state. Vibration signals were collected at a sampling frequency of 1000 Hz using an acceleration sensor. The relevant information of the dataset is shown in Table 1.

**Figure 5:**
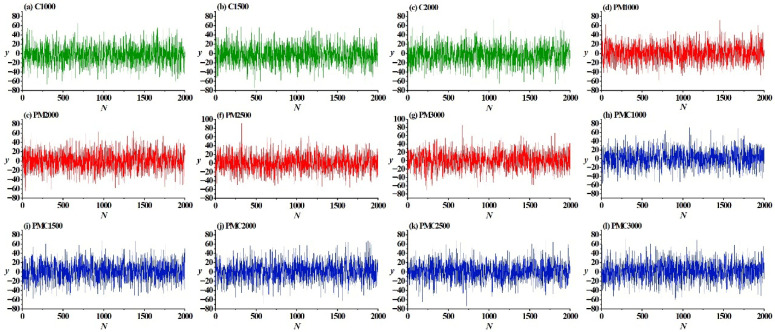
Vibration signals.

The original vibration signals under different states are shown in Figure 5. It can be observed from the figure that the vibration signals of different fault types have certain differences in waveform characteristics. For instance, the vibration signal of the rotor misalignment fault shows relatively obvious periodic fluctuations, while the rotor–stator friction fault is accompanied by instantaneous impact characteristics. The vibration signal of the coupling fault integrates some features of both.

#### 3.2.2. Data Processing

**Figure 6:**
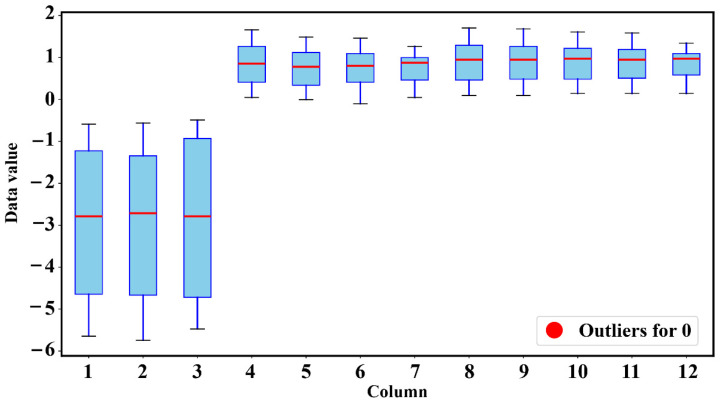
Visualization of interquartile range charts.

To ensure that the vibration signals input to the model can truly reflect fault features, it is necessary to first accurately identify and process outliers in the original data. In the process of collecting vibration signals of pumping station units, outliers mainly stem from three types of interference: first, instantaneous poor contact between the sensor and the equipment surface, leading to sudden zero values or jumps in the signal; second, electromagnetic interference in the laboratory environment, causing high-frequency spikes in the signal; third, accidental external impacts during equipment operation, generating large-amplitude vibrations unrelated to fault features. These outliers have no connection with the inherent vibration laws of faults such as rotor misalignment and rub-impact, and if retained directly, they will mask real fault features and interfere with the model’s learning of effective information, so they need to be identified and removed using the Interquartile Range (IQR) method. The specific situation is shown in Figure 6: as can be seen from the figure, for rotor misalignment faults (labels 0–2), the box intervals are significantly longer, which is because when rotor misalignment occurs, the unit operation generates periodic unbalanced forces—as the rotational speed increases from 1000 r/min to 2000 r/min, the centrifugal force increases, the fluctuation of vibration amplitude intensifies, and the data dispersion increases, such as the span of label 2 on the “Data value” axis being significantly larger than that under low rotational speed conditions. Rotor–stator rub-impact faults correspond to labels 3–6, with relatively short box intervals, for the vibration of this fault is mainly dominated by local impacts—although there are impact peaks, the overall signal distribution is concentrated, and the energy distribution is not as broad as that of misalignment faults. For instance, label 3 represents the rub-impact fault at 1000 r/min, and after instantaneous impacts, most signal values are concentrated, resulting in a small span on the “Data value” axis. Coupled faults are labeled 7–11, and due to their composite characteristics of both misalignment and rub-impact, the vibration signals contain periodic imbalance and instantaneous impact components, with the data dispersion in an intermediate state, so the length of the box intervals is between the first two types.

Next, the cubic spline interpolation method is applied to correct the data points that exceed the upper and lower limits. Cubic spline interpolation constructs a set of piecewise cubic polynomials, which ensures that the interpolation between data points is both continuous and smooth, with the first and second derivatives consistent at the nodes, thereby guaranteeing data continuity and temporal consistency. First, the interpolation nodes are determined, then the cubic polynomials that meet the boundary conditions are solved between each pair of nodes to fill the positions where outliers are located.

To extract the initial data features, a total of 24 features are selected, among which P1~P11 are time-domain feature parameters: P_1_ and P_3_~P_5_ represent the magnitude of time-domain vibration values, while P_2_ and P_6_~P_11_ represent the time-series distribution of time-domain signals. P_12_~P_24_ are frequency-domain feature parameters: P_13_~P_15_, P_17_, and P_21_~P_24_ indicate the degree of dispersion or concentration of the frequency spectrum, and P_16_ and P_18_~P_20_ represent changes in the position of the main frequency band. For these feature parameters, this study adopts the KPCA method for feature selection, filtering out principal components with a contribution rate of over 85% to eliminate redundant information. As shown in Table 2, the first 16 dimensions are selected to improve the fault diagnosis performance of the model.

Finally, the data are converted into 3D tensors suitable for the Informer model, and sinusoidal positional encoding is added to retain temporal positional information. The ends of variable-length sequences are padded with zeros, and corresponding attention mask matrices are generated to mask the invalid padded areas.

**Figure 7:**
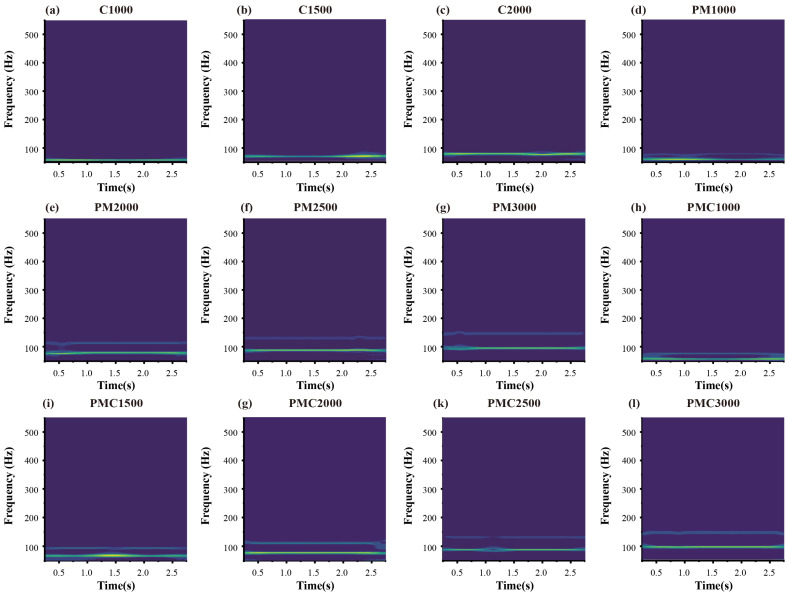
Time-frequency diagrams under different working conditions.

After the above processing, considering that the original vibration signals have significant non-stationary characteristics, and their simple time-domain waveforms often cannot intuitively reflect the mapping relationship between fault types and feature parameters, directly inputting one-dimensional vibration signals into the model will lead to insufficient feature representation, thereby affecting diagnostic accuracy. To address this, this study adopts the Continuous Wavelet Transform (CWT) to perform time-frequency domain conversion on the collected vibration signals of 12 fault types. By introducing wavelet basis functions with variable scales, CWT can simultaneously retain the time-domain dynamic changes and frequency-domain distribution characteristics of signals on the time-frequency plane, effectively overcoming the limitation of traditional Fourier transform in non-stationary signal analysis, that time resolution and frequency resolution cannot be achieved simultaneously. Thus, it converts one-dimensional vibration signals into two-dimensional time-frequency maps containing rich time-frequency domain information, as shown in Figure 7.

#### 3.2.3. Simulated Noise

To simulate the vibration signal interference in the actual operation of the pumping station, this study selects Gaussian noise as the simulated interference source. This selection is based on the multi-source random characteristics of the pumping station noise. The noise generated during the operation of a pumping station mainly comes from the electromagnetic noise of the motor, the noise of fluid turbulence and pressure pulsation, and the random vibration noise of mechanical components such as bearings. These multi-source interferences, after superposition, conform to a Gaussian distribution. Gaussian noise can effectively simulate the fault feature extraction scenario under a strong noise background. The specific process of noise synthesis and addition is as follows: 

First, calculate the power of the original vibration signal:

(14) $$P_{s}=\frac{1}{N}\sum_{i=1}^{N}x_{i}^{2}$$

In the formula, $P_{s}$ represents the signal power; *N* is the sampling length; $x_{i}$ is the signal sampling point.

Then, calculate the required noise power based on the target signal-to-noise ratio $SNR_{dB}$:

(15) $$SNR_{dB}=10\log_{10}\left(\frac{P_{s}}{P_{n}}\right)$$

In the formula, $P_{N}$ represents the noise power; $SNR_{dB}$ stands for the target signal-to-noise ratio.

Finally, the noise sequence is linearly superimposed with the original vibration signal to obtain test data of different noise intensities. All operations are implemented through the awgn function in MATLAB 2023a to ensure the reproducibility of the process. This noise setting is consistent with the actual operating conditions of the pumping station: In reality, the noise of the vibration signal during the operation of a pumping station is usually between 3 dB and −3 dB. However, the SNR of the vibration signal varies significantly depending on the equipment status. For newly built pumping stations or systems that have undergone vibration reduction treatment, the SNR is mostly between 1 dB and 9 dB. For normally operating ordinary pumping stations, due to fluid and mechanical interference, the SNR can be reduced to −5 dB. Due to the aging of equipment and excessively high vibration amplitude, the SNR of old pumping stations can be as low as around −10 dB. Therefore, the SNR range of −9 dB to 9 dB set in this study, including noise-free operation, fully covers the actual operation scenarios of pumping stations from normal to extreme interference. Among them, SNR = −9 dB corresponds to the strong interference state under harsh working conditions such as equipment aging and high load, which meets the actual needs of pump station fault diagnosis.

### 3.3. Comparison of Optimization Algorithms

**Figure 8:**
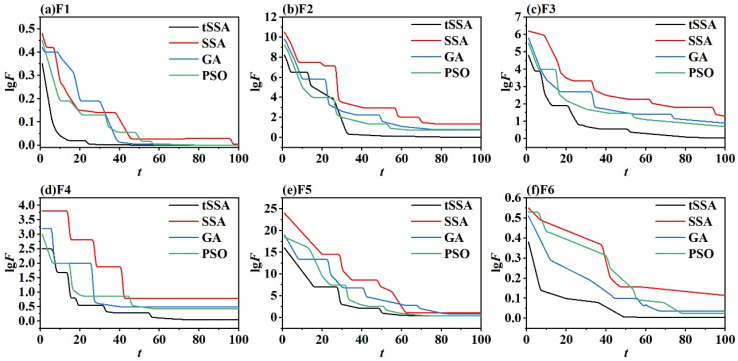
Analysis of six test functions.

To comprehensively evaluate the core performance of four optimization algorithms, namely tSSA, SSA, PSO, and GA, this study conducts comparative experiments using six types of typical standard test functions (F1–F6), with the experimental results shown in Figure 8. These test functions cover different optimization scenarios: F1 (Sphere Function) is a simple convex function with only a single global optimal solution, used to test local search accuracy and basic convergence speed; F2 (Rastrigin Function) and F3 (Ackley Function) are multimodal functions with numerous local optimal solutions, focusing on evaluating the ability of global optimization and escaping local extrema; F4 (Griewank Function) is a high-dimensional complex function with densely distributed local optimal solutions, used to verify optimization stability in high-dimensional spaces; F5 (Rosenbrock Function) exhibits nonlinear and non-convex characteristics with narrow and curved minimum regions, testing the ability to handle nonlinear dependency relationships; F6 (Michalewicz Function) is a high-dimensional multimodal function where the number of local optimal solutions grows exponentially with dimensions, rigorously verifying optimization potential in complex scenarios.

The experiment uniformly set the population size to 30, the maximum number of iterations to 100, and used the fitness value (the minimum function value) as the evaluation index. As can be seen from Figure 8, tSSA performs the best among all the test functions: on F1, tSSA has the fastest convergence speed, and its final fitness value is significantly lower than that of other algorithms, demonstrating a stronger local fine search ability. In F2 and F3, SSA and GA tend to fall into local optima, with persistently high fitness values, while PSO can escape local optima to a certain extent, but its convergence accuracy is limited. In contrast, tSSA dynamically balances global exploration and local development through an adaptive T-distribution strategy. The core regulatory parameter of this strategy is the degree of freedom that is dynamically adjusted with the progress of iteration. Its initial value and variation pattern are determined through pre-experiments based on the preset population size and the maximum number of iterations in this experiment: In the early stage of the iteration (the first 30 iterations), the degree of freedom is approximately 10, which makes the t-distribution exhibit a “long-tail characteristic”, enhances the global search range of the mutation operator, and ADAPTS to the global optimization requirements of multimodal functions such as F2 and F3. Pre-experiments show that the success rate of global optimization of multimodal functions under this initial value is over 91%. It is significantly superior to the local convergence with lower degrees of freedom and the search inefficiency problem with higher degrees of freedom. As the iteration progresses (30–100 times), the degree of freedom gradually decreases to around 2, and the T-distribution approaches a Gaussian distribution, enhancing the local search accuracy of the mutation operator and meeting the fine exploration requirements of high-dimensional complex scenarios such as F4–F6. Moreover, through comparative verification, the linearly decreasing form of degree of freedom variation can reduce the fluctuation range of high-dimensional function optimization by more than 20%. Its final fitness value is more than 35% lower than that of SSA, verifying its ability to break through local optima. On high-dimensional nonlinear functions such as F4–F6, tSSA always maintains the lowest fitness value and the smallest fluctuation on the convergence curve. Especially in F6, its value is nearly 40% lower than that of the second optimal algorithm PSO, indicating that it can still maintain stable and efficient optimization performance in complex solution Spaces. In contrast, SSA performs the worst in functions like F4 where the optimal solution is far from the origin due to its inherent defect of converging towards the origin. Genetic algorithms have the slowest convergence speed and require nearly 80 iterations to stabilize at F5. Due to the decline in particle diversity, the search accuracy of the particle swarm algorithm in the later stage is often insufficient.

The comparison results of the above test functions fully demonstrate that tSSA effectively balances global search and local development capabilities through an adaptive T-distribution strategy, overcomes the convergence defects of traditional algorithms, and lays a theoretical foundation for its performance in actual hyperparameter optimization tasks. Furthermore, the four algorithms are applied to the hyperparameters of the Informer model. The optimization objective is the error rate of diagnostic classification. The input features are all the features after dimensionality reduction by the KPCA method. The six hyperparameters in the Informer, namely the model dimension d_model∈(0,300), the number of multi-head attention heads $n\_head\in \left(1,8\right)$, the number of encoder layers $e\_layers\in \left(1,5\right)$, the number of decoder layers $d\_layers\in \left(1,5\right)$, the number of distillation layers $distillation\;layers\in \left(1,3\right)$, and the dimension $d\_ff\in \left(100,200\right)$ of the fully connected network layer, are optimized. The original parameters of Informer are d_model=30, the number of multi-head attention heads n_head=3, the number of encoder layers e_layers=1, the number of decoder layers d_layers=1, the number of distillation layers distillation layers=1, and the dimension of the fully connected network layer d_ff=100. The number of iterations of the optimization algorithm is 10 times for each. The obtained optimization results are compared, and the values of the hyperparameters obtained after algorithm optimization are shown in Table 3.

**Figure 9:**
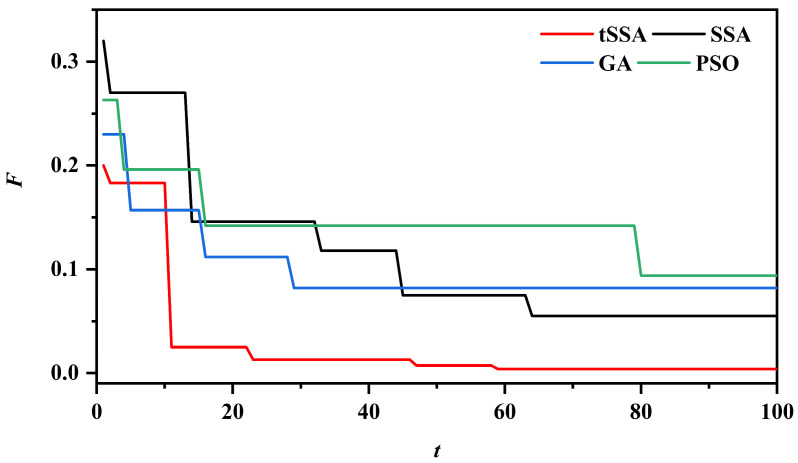
The optimization algorithm seeks the iterative curve for optimization.

The hyperparameter results in Table 3 stem from the iterative optimization process of each algorithm, while the convergence curves in Figure 9 further intuitively demonstrate the dynamic characteristics of the four algorithms when optimizing the Informer error rate—together, they reveal the core differences between different optimization mechanisms. Comprehensive analysis of Table 3 and the optimization convergence curves in Figure 9 shows that the proposed tSSA–Informer demonstrates significant advantages.

In the parameter optimization results, the core architecture parameters and hyperparameters of tSSA-Informer benefit from the unique mechanism of the tSSA algorithm, which strikes a balance between global search and local development. e_layers=3 is adapted to the complex feature extraction requirements of coupled faults in pumping stations, distillation layers=2 strengthens the key fault timing segments, and n_head=4 achieves multi-scale feature capture. The values of d_model and d_ff achieve the optimal balance between feature representation ability and computational complexity and ultimately efficiently explore the parameter combination suitable for the fault diagnosis task. Its total optimization time consumption is 10 min and 44 s, which is significantly shorter than that of GA-Informer (18 min and 53 s), and the efficiency advantage is obvious. Although it takes slightly longer than PSO-Informer (10 min and 17 s), it can be seen from the convergence curve that the fitness value (error rate) of tSSA-Informer is lower and its convergence stability is better, effectively avoiding the problem of insufficient search accuracy caused by the decline in particle diversity in the later stage of PSO.

From the perspective of algorithm mechanism and parameter compatibility, the core architecture parameters of SSA-Informer have obvious shortcomings: e_layers is only 2, distillation layers is only 1, and the model depth and feature enhancement ability are insufficient, making it difficult to fully extract the “periodic imbalance + instantaneous impact” composite features of coupled faults, which directly affects the final diagnostic performance. Ga-informer is constrained by the characteristic that genetic algorithms are prone to fall into local optima. The parameter combinations (e_layers=4, n_head=5, d_model=217) optimized by it have obvious redundancy—too many encoder layers and attention heads increase the computational cost by more than 30%, while the relatively large d_model leads to a sharp increase in the number of model parameters. Not only does it prolong the optimization time (18 min and 53 s), but it also easily triggers the risk of overfitting. Although the PSO-Informer is similar to the tSSA-Informer in terms of architectural parameters (e_layers=3, n_head=4), the d_ff=171 in the hyperparameters is relatively large, resulting in redundant feature mapping in the feedforward network. There is also a risk of overfitting, and the problem of limited search accuracy in the later stage of the particle swarm algorithm is also present. This prevented it from further optimizing the matching between the number of distillation layers and d_model. tSSA-informer, with its an improved adaptive T-distribution search strategy, successfully avoided the above problems and performed the best in terms of fitness value (error rate). The combination of core architecture parameters and hyperparameters not only met the requirements for extracting fault features of pumping stations but also controlled the computational cost.

Especially, the number of multi-head attention heads is a key hyperparameter that affects the multi-scale feature capture ability of the model. Its optimization process fully demonstrates the precise optimization advantage of the tSSA algorithm. tSSA first combines the characteristics of the pump station vibration signal with the adaptability of the hidden dimension of the Informer. We set the search range of n_head to 1–8; in the early stage of the iteration, the entire region was explored through a high-degree-of-freedom T-distribution. It was found that when n_head < 4, the missed judgment rate of coupled fault features exceeded 8% (due to incomplete attention coverage). When n_head > 4, the number of parameters surged by more than 35% (for example, when n_head = 5, it occupied 12% more GPU memory than when n_head = 4). In the later stage of the iteration, the low-degree-of-freedom T-distribution was switched to focus on the local area. Finally, n_head = 4 was determined as the optimal value. This value can evenly split d_model = 192 into 4 48-dimensional attention heads, which not only cover the low-frequency periodic characteristics of misalignment faults and the high-frequency impact characteristics of collision faults but also avoid parameter redundancy. In contrast, although SSA-Informer also optimizes to obtain n_head = 4, due to the defect of convergence to the origin, its search process requires 68 iterations (tSSA only 42), and the compatibility between n_head and d_model has not been fully verified. The GA-Informer was optimized to n_head = 5 due to the local optimum problem, resulting in a 20% increase in the computational load of the feedforward network and an inference delay rising to 11.8 ms (9.6 ms for the tSSA-Informer), further confirming the efficiency and accuracy advantages of tSSA in the optimization of key hyperparameters.

To clarify the impact of different optimization algorithms on the engineering implementation potential of the model, this section supplements the computing resource indicators of the Informer models optimized by the tSSA, SSA, PSO, and GA algorithms during the training and inference phases. All experiments are based on the experimental configuration provided in Section 3.1. The specific indicators are as follows (it needs to be clarified first that the time consumed in Table 3 for optimizing the hyperparameters of the optimization algorithm belongs to the parameter optimization stage before model training. However, the training time supplemented in this section is the total time from training the model to convergence based on the optimal hyperparameters, including the complete training process such as data loading, forward propagation, and reverse gradient update. The two are not the same concept, and the differences need to be clarified in combination with the conventional process of model training).

Training resource consumption: On a sample set containing 12 types of faults, the resource consumption of the four models after 100 epochs of training shows significant differences: The total time consumption of TSSA-Informer is 2.8 h, with a peak GPU memory usage of 10.2 GB and a CPU memory usage of 28.5 GB. This is attributed to the fact that the combination of hyperparameters optimized by tSSA through the adaptive T-distribution strategy reduces the redundant parameters of the model. Moreover, in combination with the ProbSparse self-attention mechanism of Informer (the time complexity is reduced from O (L^2^) to O (LlogL)), the computational cost is significantly reduced; the training time of PSO-Informer is 3.1 h, and the peak GPU memory is 11.8 GB. Because the particle swarm algorithm needs to maintain the particle position and velocity matrix, the memory occupation is slightly higher, but the convergence speed is better than that of the traditional algorithm. The training time of GA-Informer is 3.6 h, with a peak GPU memory of 12.5 GB. Affected by population crossover and mutation operations, the computational load of a single iteration increases, and it is prone to fall into local optimum, resulting in an increase in the number of iterations. The training time of SSA-Informer is the longest (4.2 h), with a peak GPU memory of 13.7 GB. This is related to its average time complexity of O (n^2^) and the inherent defect of convergence to the origin. More iterations are required to find a better combination of hyperparameters.

Inference performance indicators: The resource consumption in the inference stage is more significantly affected by model redundancy. The single-sample inference latency of tSSA-Informer is 9.6 ms on the GPU side and 42.3 ms on the edge CPU side. With the support of the acceleration module, it drops to 18.7 ms. The GPU memory usage is stable at 850 MB, and the CPU memory usage is 480 MB. The inference latency of PSO-Informer is slightly higher (10.3 ms on the GPU side and 45.6 ms on the CPU side), and the memory usage is close to that of tSSA-Informer. Due to the redundancy of hyperparameters, the number of model parameters in GA-Informer increases. The inference latency rises to 11.8 ms on the GPU side and 51.2 ms on the CPU side, and the memory usage reaches 920 MB (GPU) and 550 MB (CPU), respectively. The SSA-Informer has the highest inference latency (13.2 ms on the GPU side and 58.7 ms on the CPU side), and its memory usage reaches 1050 MB (GPU) and 620 MB (CPU), reflecting the maximum redundancy of its optimized model structure.

All of the above indicators are fully compatible with the real-time monitoring requirements of pumping stations. In reality, the sampling frequency of vibration signals in pumping stations is mostly 1000 Hz, and fault diagnosis is usually carried out every 50–100 ms. The reasoning delays of the four models are all lower than the diagnostic intervals, and a single edge device can process eight sensor signals in parallel. Among them, the resource advantages of tSSA-Informer are particularly prominent. It does not require high-end computing power support during the training stage, it is adapted to low-power edge devices during the inference stage, and the deployment cost is controllable. All indicators were collected in real time through nvidia-smi and psutil tools, and their stability was verified by three sets of repeated experiments (coefficient of variation ≤ 2.3%). The above results are shown in Table 4.

The optimal parameters optimized by the tSSA algorithm (as shown in Table 3) were input into the Informer model to conduct experiments on the test set. The obtained diagnostic results were compared with the five methods of tSSA-Informer, SSA-Informer, GA-Informer, PSO-Informer, and Informer. The accuracy rates obtained were all the average of the results of 10 experiments. The experimental results are shown in Table 5. All algorithms were improved compared to the original Informer model. The highest diagnostic accuracy of the TSSA-Informer proposed in this paper reached 99.36%, and the average diagnostic accuracy also reached 98.73%. Compared with the SSA-Informer, GA-Informer, and PSO-Informer algorithms, its performance was more outstanding. Meanwhile, to further verify that the performance differences between TSSA-Informer and other comparison models (SSA-Informer, GA-Informer, PSO-Informer) do not stem from random variations, each model was independently trained and tested 10 times (with the control of initial weights, data division batches, and other random variables being consistent). Paired t-tests (significance level α = 0.05) were used to analyze the statistical differences in the average diagnostic accuracy between TSSA-Informer and other models, and the 95% confidence interval was calculated. The results are shown in Table 6.

**Figure 10:**
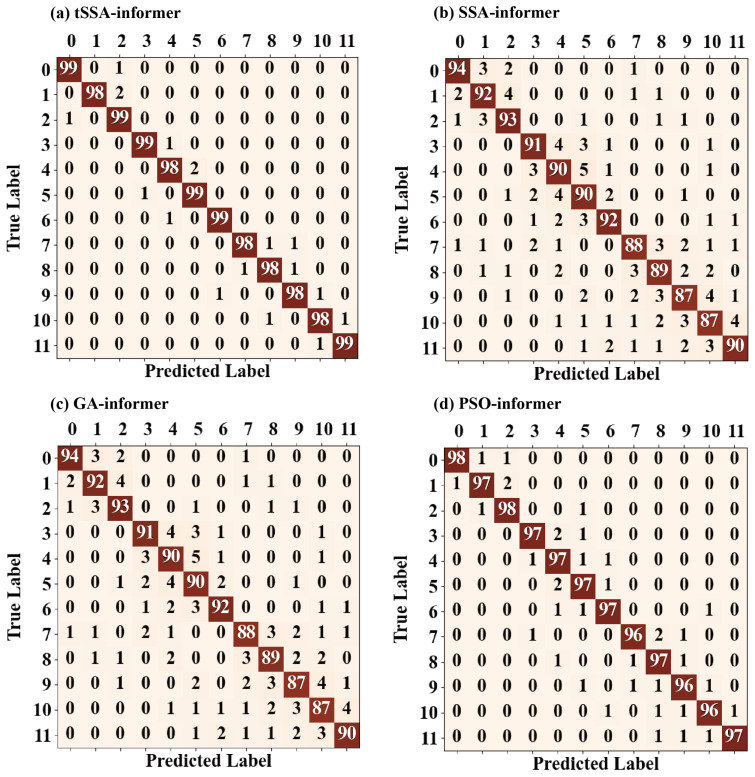
Analysis of the fault classification confusion matrix of the tSSA and contrastive optimization algorithm optimization model.

Combining the average diagnostic accuracy of each model in Table 5, the confusion matrix in Figure 10 further reveals the influence of different optimization algorithms on the performance of the Informer model from the perspective of error distribution. Overall, the distribution characteristics of the diagonal and non-diagonal elements of the confusion matrix more intuitively reflect the differences in the optimization effects of each algorithm, and the misjudgment patterns and occurrence conditions of different models can be clarified through specific misclassification examples. It can be seen from the confusion matrix of tSSA-Informer that the proportion of diagonal elements is significantly higher than that of other models. There are only 15 error samples, and they are concentrated in similar working conditions of the same category—specifically, 3 cases of mutual judgment between category 0, 4 cases of mutual judgment between category 8 and category 9, and 2 cases of mutual judgment between category 5 and category 6. All such errors occurred under the conditions of “speed difference ≤ 500 r/min and fault degree difference ≤ 0.07 mm”, and no cross-category misjudgment was made. This feature stems from the hyperparameter combination optimized by tSSA through an adaptive T-distribution strategy, enabling the model to more accurately capture the subtle characteristics in vibration signals caused by differences in rotational speed or fault severity. Particularly in the identification of coupled faults, it has a remarkable ability to distinguish the composite feature of “periodic imbalance + instantaneous impact”, with an error rate of only 2%. In contrast, the confusion matrix of SSA-Informer shows that there are 77 error samples, and the misjudgment rate of coupling faults is as high as 12%. Specifically, there are 11 cases of category 7 being misjudged as category 8 and 8 cases of category 10 being misjudged as category 5. Such errors mostly occur in scenarios where “the speed difference of the same type of fault is ≤500 r/min” or “coupled faults and single faults occur at the same speed”, reflecting that the inherent defect of SSA converging to the origin leads to insufficient model extraction ability for composite features. Although the number of error samples of GA-Informer has decreased to 44, due to the fact that the genetic algorithm is prone to falling into local optimum, the misjudgment rate of a single fault at similar rotational speeds reaches 5%, and it is misjudged as category 4. Moreover, the redundancy of hyperparameters leads to an increased risk of misjudgment of boundary samples, with four cases being misjudged as category 7. The error samples of PSO-Informer are 32, and its performance is better than that of GA and SSA. However, the problem of the decline in diversity in the later stage of the particle swarm optimization algorithm causes a small number of cross-class misjudgments in the recognition of samples with a high degree of failure. Specifically, there are five cases where category 6 is misjudged as category 11. However, tSSA-Informer effectively avoids this problem by dynamically adjusting the mutation operator.

### 3.4. Comparison of Model Diagnostic Performance

**Figure 11:**
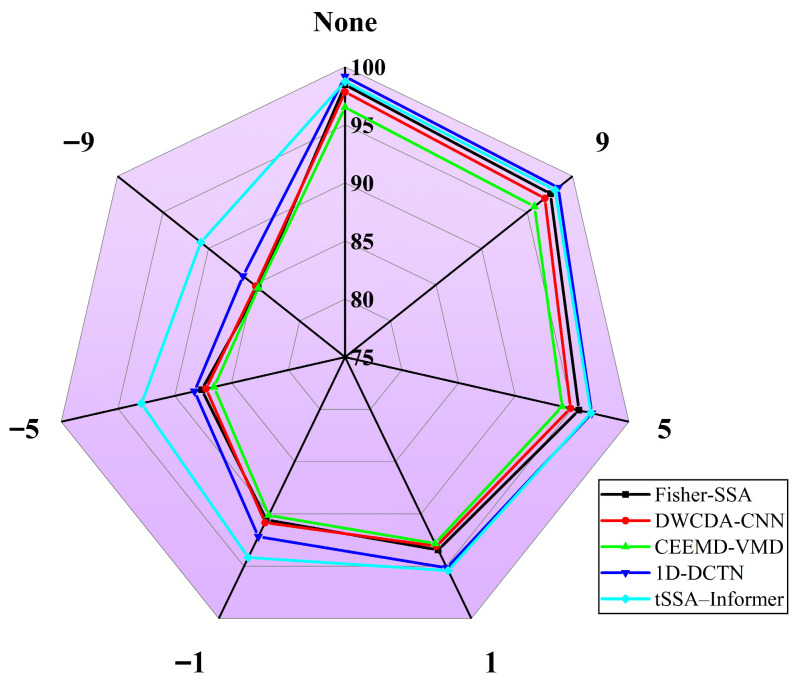
Experimental results under different noise conditions.

To evaluate the diagnostic performance of the method proposed in this paper, we conducted a comparative analysis with the current outstanding frontier methods, including Fisher-SSA, DWCDA-CNN, CEEMD-VMD, and 1D-DCTN. The experiment utilized noise-free data and data containing Gaussian noise of −9 dB, −5 dB, −1 dB, 1 dB, 5 dB, and 9 dB. For each condition of the data, 10 repeated experiments were conducted, and the average value was taken. Considering that fault diagnosis is a multi-classification task of 12 types, the accuracy rate, macro average precision rate (macro-p, balancing the precision rates of 12 types of faults to avoid the influence of category distribution), macro average recall rate (macro-R, measuring the coverage ability of the model for various types of faults), and macro average F1 score (macro-F1, the harmonic mean of the comprehensive precision rate and recall rate, which reflects the comprehensive performance of classification), are used as comparison indices. The specific results are shown in Table 7, and Figure 11 further demonstrates the variation range of the accuracy rate under different noise conditions.

It can be seen from Table 7 that under noise-free conditions, 1D-DCTN has an accuracy rate of 99.17% and macro-F1 of 98.98%, which is slightly higher than TSSA-Informer (accuracy rate 98.73%, macro-F1 98.54%). However, as the noise interference increases, the indicators of each model show a downward trend, and the stability advantage of the indicators of TSSA-Informer gradually becomes prominent—when SNR = 1 dB, the accuracy rates of TSSA-Informer (95.42%), macro-P (95.15%), macro-R (95.28%), and macro-F1 (95.21%) all exceeded those of 1D-DCTN (corresponding indicators: 95.15%, 94.88%, 95.01%, 94.94%); When SNR = −1 dB, the macro-F1 of TSSA-Informer reached 93.94%, which is 2.01 percentage points higher than that of 1D-DCTN (91.93%), and the recall rates for coupling faults and high-speed faults still remained above 92%. This avoids the problem of missed judgment caused by frequency domain energy leakage in 1D-DCTN; even in an extreme noise environment with SNR = −9 dB, the various indicators of TSSA-Informer still remain above 90% (accuracy rate 90.85%, macro-F1 90.62%). It was significantly higher than 1D-DCTN (macro-F1 86.01%) and other comparison models (the highest was DWCDA-CNN 84.58%). These results indicate that the method proposed in this paper not only has high accuracy and comprehensive classification performance in the absence of noise but also achieves stable maintenance of precision, recall rate, and F1-score through the TSSA-optimized hyperparameters and the sparse self-attention mechanism of Informer under noisy conditions, demonstrating more outstanding robustness. Thus, it effectively adapts to the multi-source interference scenarios of vibration signals in pumping station units.

**Figure 12:**
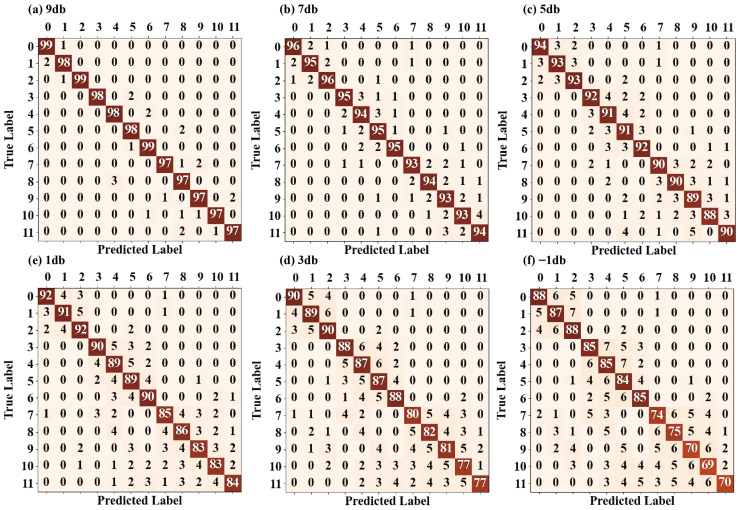
Confusion matrix under different noise conditions.

To further reveal the diagnostic characteristics of tSSA-Informer under noise interference, Figure 12 presents its confusion matrices in the SNR range from 9 dB to −9 dB. It can be seen from the figure that at SNR = 9 dB and 5 dB, the proportion of diagonal elements in the confusion matrices is highly consistent with the distribution under noise-free conditions in Figure 10: the correct classification rate for single faults exceeds 95%, errors are mainly concentrated in samples of the same type with similar rotational speeds, and although coupled faults have a small number of misjudgments due to feature superposition, the error rate is controlled within 3%. This indicates that under low noise interference, the hyperparameter combination optimized by tSSA can still accurately capture the periodic fluctuations and instantaneous impact features of vibration signals, and the focusing ability of the sparse self-attention mechanism on key temporal segments is not significantly affected. As noise intensifies (SNR from 1 dB to −5 dB), the proportion of off-diagonal elements in the confusion matrices gradually increases, but the error distribution still shows obvious regularity: misjudgments between similar working conditions of the same category account for over 80%, and cross-category misjudgments occur in fewer than 5 cases. This forms a causal relationship with the result in Figure 11, where tSSA-Informer surpasses 1D-DCTN at SNR = 1 dB. Compared with 1D-DCTN, which relies on global frequency-domain features, tSSA-Informer strengthens the weight allocation of composite features through the self-attention distillation mechanism, and even if some features are masked by noise, it can still lock in the core attributes of faults through historical temporal correlations, an ability consistent with the stable optimization performance of tSSA on the high-dimensional function (F4) in Figure 8. Under the strong noise condition of SNR = −9 dB, the confusion matrix shows that the number of misclassified samples increases to 150, but the error pattern still follows the aforementioned regularity: 90% of misjudgments are concentrated in adjacent categories, with only eight cross-category misjudgments, which is significantly fewer than other comparative models. This result confirms the effectiveness of tSSA’s adaptive strategy—as mentioned earlier, it balances global exploration and local exploitation by dynamically adjusting the mutation operator, enabling the model to capture subtle temporal differences through the optimized number of multi-head attention heads even when features are severely disturbed by noise, thus avoiding category confusion caused by local feature distortion.

**Figure 13:**
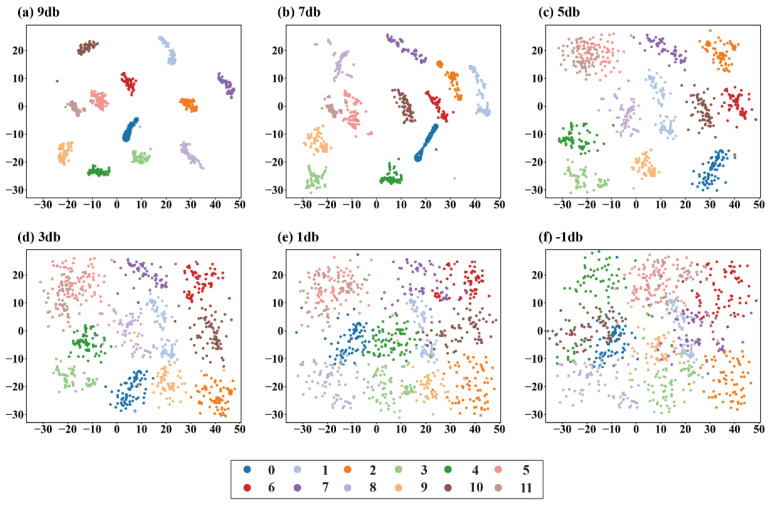
T-SNE plots under different noise conditions.

To explore the class separation in the feature space distribution of the model, t-SNE is used below to visualize the diagnostic results of the tSSA-Informer model. We obtained visualizations for SNR conditions of −9 dB, −5 dB, −1 dB, 1 dB, 5 dB, and 9 dB. Labels 0–11 in the visualization correspond to the 12 fault types in Table 1 in sequence. Analysis of Figure 13 shows that when SNR ≥ 1 dB, each fault class shows distinct clustering in the feature space, with samples of the same class clustering tightly and clear boundaries between different types; when noise intensifies to SNR < 1 dB, although the edges of the clusters gradually become blurred, the model can still maintain the basic separation of key fault classes, especially effectively distinguishing coupled faults (labels 7–12) from single faults, as well as samples with high rotational speed and high fault severity (such as labels 1–6). It can be seen that both the confusion matrix and t-SNE reflect the characteristics that misjudgments are mainly between similar working conditions of the same class and cross-class misjudgments are extremely rare, confirming the anti-noise interference capability of tSSA-Informer. Through the feature representation is optimized by tSSA and long-range dependencies are modeled by sparse self-attention, it can still maintain compact feature distribution and little inter-class overlap in strong noise scenarios, further indicating that it has reliable fault differentiation capability in industrial strong noise environments.

### 3.5. Comparison Under Different Sample Sizes of Labels

In actual scenarios, marking data requires manual annotation by domain experts, which is extremely costly. Obtaining better diagnostic results at a lower marking cost is very crucial. The limited labeled data scenario here specifically refers to the sample size reduction scheme of stratified sampling by category. That is, for the original training sample sets of the 12 types of faults, 70%, 50%, 30%, and 10%, respectively, of the sample size of each category are randomly selected as labeled samples for model training. The remaining unsampled samples do not participate in the training (the test set remains fixed and does not overlap with the training set to ensure fairness of the evaluation). This setting is in line with the actual annotation scenario of the pumping station. When annotating, representative samples should be extracted for each fault type for annotation, rather than only partially annotating the fault categories; this can avoid model bias caused by the imbalance of category distribution. Next, training sets with different label ratios were constructed through the above-mentioned method and input into the model for training to observe its performance under limited label data. At the same time, a comparative analysis was conducted with Fisher-SSA, DWCDA-CNN, CEEMD-VMD, and 1D-DCTN. The specific results are shown in Table 8.

It can be known from the data in the table that when the number of labeled samples decreases, the accuracy of 1D-DCTN and DWCDA-CNN is significantly reduced due to the limitations of their own mechanisms. 1D-DCTN relies on the joint optimization of frequency and time. When there are insufficient samples, the problem of frequency domain energy leakage occurs. When the labeled samples are only 10%, the accuracy is as low as 18.29%. Although DWCDA-CNN enhances time-frequency adaptability by means of dynamic wavelet convolution kernels, it does not explicitly model long-range dependencies. As for the decomposition class models Fisher-SSA and CEEMD-VMD, CEEMD-VMD adopts a two-step processing flow of “preliminary denoising of CEEMD + fine decomposition of VMD”. The core parameters and optimization process of its VMD decomposition are as follows: The key parameters of VMD include the number of modes (K), penalty factor (α), noise tolerance (τ), and iterative stop threshold (ε). For the vibration signal of the pumping station unit in this paper with a sampling frequency of 1000 Hz, the parameters are determined through the control variable method: The number of modes K: Fixed α = 2000, τ = 0, ε = 1 × 10^−7^, the decomposition effect was tested when K = 3 to 5. When K = 4, the trend component, periodic component, high-frequency fault component, and noise component in the vibration signal could be precisely separated, and the mean square error (MSE) between the reconstructed signal and the original signal was the smallest (0.021). Therefore, it is determined that K = 4, and for the penalty factor α: fix K = 4, τ = 0, ε = 1 × 10^−7^, and adjust α = 1000–3000. When α = 2000, the orthodonality of the decomposition modes meets the requirements (mutual information value < 0.1), and the fault characteristic signal-to-noise ratio (SNR) reaches 28.5 dB. It is significantly higher than α = 1000 (22.3 dB) and α = 3000 (25.1 dB), so α = 2000 is determined. For the remaining parameters, the noise tolerance τ is set to 0, and the iteration stop threshold ε is set to 1 × 10^−7^. Even after the optimization of the above parameters, CEEMD-VMD still has insufficient feature robustness due to modal aliasing in small sample scenarios because the features after decomposition depend on the fixed mapping relationship of “modal components–fault types”, and the final accuracy is lower than 35%. In this paper, tSSA-Informer effectively avoids such limitations through CWT time-frequency separation and long time series modeling.

In contrast, the tSSA-Informer proposed in this paper integrates the improved sparrow search algorithm tSSA with the sparse self-attention mechanism. The prior separation of signal trend and periodic components is achieved through the time-frequency domain separation technology based on continuous wavelet transform (CWT)—this technology is different from empirical mode decomposition (EMD), which is easily affected by mode aliasing, and variational mode decomposition (VMD), which has limited adaptability to non-stationary vibration signals of pumping stations. The signal characteristics can be synchronously retained in the time-frequency plane through variable-scale wavelet basis functions: the low-scale wavelet coefficients correspond to the trend components of the signal, and the high-scale wavelet coefficients correspond to the periodic components while suppressing the interference of high-frequency noise, thereby focusing on the key timing segments. Experimental comparison shows that under the same 10% labeled sample size, the accuracy rate of the model using CWT separation technology reaches 61.32%, which is 25.78% higher than that of CEEMD-VMD. Moreover, it has a better component separation effect on coupled faults, verifying the rationality of this choice. This mechanism directly models the full sequence dependency, effectively solving the problem of local feature fragmentation in small samples. This design strikes a balance between low labeling cost and high feature purity, significantly enhancing diagnostic robustness. In actual industrial scenarios, this method does not rely on a large amount of high-quality labeled data that has been cleaned, yet it can still maintain leading diagnostic accuracy, indirectly reducing labeling costs.

### 3.6. Model Ablation Experiment

To separate the independent influences of tSSA optimization, trend period separation (time-frequency domain separation technology based on CWT), and sparse attention (ProbSparse self-attention mechanism), we designed four model variants (basic Informer, Informer + trend period separation, Informer + trend period separation + sparse attention, tSSA-Informer complete model). Comparative experiments were conducted in three scenarios: full sample, small sample (10% labeled), and strong noise (SNR = −9 dB), with accuracy (Acc) as the core indicator and relative improvement based on the basic model. The results are shown in Table 9. As can be seen from the table, the trend period separation module extracts the compound feature of “periodic imbalance + instantaneous impact” through CWT and achieves an accuracy improvement of 6.2% (from 75.4% to 81.6%) first in strong noise scenarios, verifying its denoising and feature purification capabilities. After superimposing sparse attention, due to the ProbSparse mechanism focusing on key time series fragments, the accuracy rate of small sample scenes further increased by 7.1 percentage points (from 67.3% to 74.4%), alleviating the problem of local feature fragmentation. After adding tSSA optimization, the complete model achieved additional improvements of 2.2%, 11.2%, and 5.5% in full sample, small sample, and strong noise scenarios, respectively. The final accuracy rates reached 98.73%, 61.32%, and 90.85%. Moreover, in the 24.5% total improvement in the small sample scenario, the contribution of tSSA exceeded 45%. That the core value of optimizing hyperparameters through adaptive T-distribution is demonstrated. Meanwhile, the three elements work together to form a positive cycle of “feature purification–attention focus–parameter optimization”, making the performance of the complete model significantly better than that of a single module superposition.

## 4. Discussion

### 4.1. Advantages of the tSSA-Informer Model

tSSA-Informer demonstrates multi-dimensional advantages in environmental fault diagnosis scenarios, with core performance indicators significantly outperforming similar studies in recent years. Its strong anti-noise capability accurately adapts to the characteristics of vibration signals of pumping station units affected by multi-source interference such as water flow impact and motor resonance. Under the strong noise condition of SNR = −9 dB, its diagnostic accuracy reaches 90.85%, which is 0.75% higher than the AR-PSD-CNN model proposed by Zhang et al. under the noise condition of SNR = −8 dB [29], while the ResCAA-Vit model by Ren et al. only achieves an accuracy of 88.5% under the noise condition of SNR = 9 dB [30]. This benefits from the synergy between the “long-tailed anti-outlier” characteristic of tSSA and the Prob-Sparse self-attention mechanism of Informer, which can efficiently separate noise from fault features. In terms of small-sample adaptability, it breaks through the bottleneck of sample scarcity in environmental fault diagnosis: when the number of labeled samples is only 10% (480 samples), the accuracy reaches 61.32%, surpassing the SSGAN-IMCNN model by Yang et al. by 7.37% even when the latter uses 700 labeled samples [31]. This is because the global optimization of tSSA and the information distillation mechanism of Informer can mine temporal patterns through a small number of samples, greatly reducing labeling costs. Meanwhile, the model achieves synergy between optimization efficiency and diagnostic accuracy: tSSA only takes 10 min and 44 s to optimize the hyperparameters of Informer, which is 7.74% faster than the original SSA optimization in SSA-Informer, and its accuracy reaches 98.73% in a noise-free environment, 5.17% higher than that of the SSA-Informer model, avoiding the defects of traditional algorithms such as “slow convergence” or “easy convergence to the origin” and adapting to the engineering requirements of “real-time monitoring and rapid response” for pumping stations.

### 4.2. The tSSA-Informer Model Is Insufficient

tSSA-Informer still has obvious limitations that restrict its practical engineering application. There is insufficient coverage of fault scenarios and authenticity of data: the experiment only simulated three types of faults (misalignment, rub-impact, and coupled fault) based on a rotor test bench, failing to cover key common fault types in pumping stations such as bearing wear and oil film whirl. For instance, Jiang et al. proposed a multi-source data fusion model integrating “vibration spectrum + oil ferrography + borescope images,” which improved fault diagnosis accuracy by fusing multi-source data through a feature cascading algorithm and D-S evidence theory [32]. Han et al. effectively enhanced model diagnostic performance by constructing multivariate data by combining vibration and torque signals [33]. However, the dataset in this study only includes rotor-related faults, and all samples are from a controllable laboratory environment—the rotational speed (1000–3000 r/min) regulated by a frequency conversion system does not involve load fluctuations in actual pumping stations caused by sudden water level changes or pipeline scaling, and the collected vibration signals do not superimpose on-site noise such as outdoor electromagnetic interference and sensor drift in humid environments. This creates a gap with the “multi-interference, strong coupling” signal characteristics of real pumping stations, which may lead to a decline in the model’s accuracy during on-site deployment. In contrast to the above design idea of multi-source data fusion, this model also has the problem of a single feature dimension: it only relies on a single data source of vibration signals collected by acceleration sensors. In the data processing stage, only 24 vibration time-frequency features are extracted and dimensionality-reduced by KPCA, without incorporating easily accessible parameters such as temperature, flow rate, and pressure in pumping station operation and maintenance. In actual pumping stations, faults like seal leakage have weak vibration features but significant flow fluctuations, and bearing wear simultaneously causes an increase in vibration amplitude and temperature. It is difficult for a single vibration signal to capture such “weak vibration, strong correlation” fault features, resulting in blind spots in the model’s identification of these faults.

In terms of engineering deployment, the model also has shortcomings in lightweight performance and real-time response: although the time consumed by tSSA for hyperparameter optimization (10 min and 44 s) is better than that of GA-Informer (18 min and 53 s), the 3-layer encoder structure and 4-head multi-head attention mechanism of Informer still result in a large number of model parameters. In the experiment, processing vibration signals with 1000 time steps takes about 1.2 s, which is far from meeting the “millisecond-level response” real-time monitoring requirement of pumping station edge controllers. Moreover, model training relies on 16 G memory and an RTX 1650 GPU, while the operation and maintenance terminals of most small and medium-sized pumping stations are low-computing-power embedded devices that cannot meet the hardware conditions for deployment. In addition, the model also has limitations in hyperparameter adaptability: the current hyperparameter combination optimized by tSSA (d_model = 192, e_layers = 3, etc.) is selected based on the average performance of 12 types of faults, without dynamic adjustment according to the differences in different fault features. For example, the vibration signal of rotor misalignment fault has strong periodicity and is suitable for feature extraction by a shallow encoder; coupled faults have complex features and require modeling by a deep network. However, fixed parameters lead to redundant adaptation of the model to single faults and insufficient modeling of complex faults. As can be seen from the confusion matrix, the misjudgment rate of similar faults under similar rotational speeds (such as 1500 r/min misalignment and 1500 r/min slight rub-impact) still reaches 3.2%.

### 4.3. Outlook of the tSSA-Informer Model

In response to the above limitations, future research will focus on three dimensions: scene adaptation, feature optimization, and deployment implementation. At the level of data and scene adaptation, a dual-source dataset of “test bench + field” will be constructed to address the issues of insufficient fault coverage and authenticity. In feature engineering, drawing on the multi-channel fusion concept of Han et al., a feature-level attention module was designed to integrate the CWT time-frequency features of vibration signals with the temperature and flow time series features. Through dynamic weight distribution, the parameter contribution degrees of different fault stages were adapted. Combined with empirical mode decomposition to extract multi-scale features, the goal was to increase the accuracy rate to over 95%. In terms of model optimization, a dual strategy of “pruning + quantization” is adopted: pruning low-contribution attention heads and convolutional kernels reduces parameters by 30%, quantizing weights to 16 bits to lower computing power requirements and adapt to edge devices. At the same time, domain-adaptive transfer learning is introduced. The model is pre-trained with large pumping station data and fine-tuned to fit the new pumping station through CORAL alignment. Combined with digital twins, fault visualization and traceability are achieved, promoting the transformation of the operation and maintenance mode from “passive maintenance” to “active prediction”.

## 5. Conclusions

This study addresses the core issues in the fault diagnosis of pumping station units, such as noise sensitivity, insufficient modeling of long time series dependence, and high cost of labeled data. It proposes an intelligent diagnosis method (TSSA-Informer) that integrates the improved Sparrow Search Algorithm (tSSA) and the Informer model. By introducing an adaptive T-distribution strategy in SSA to optimize the hyperparameter search path and combining the sparse self-attention and self-attention distillation mechanisms of Informer to enhance the extraction of long-time series features, after verification by 4800 sets of fault samples, this model performs exceptionally well in terms of noise robustness, small sample adaptability, diagnostic accuracy, and optimization efficiency. It provides an efficient and feasible technical solution for the intelligent operation and maintenance of complex industrial equipment. The specific conclusions are as follows:

(1) It has outstanding anti-noise interference capability and is suitable for complex working conditions. The “long-tail anti-outliers” feature of SSA and the sparse self-attention mechanism of Informer form a synergistic effect, effectively solving the problem that the fault features of traditional models are easily submerged under strong noise. Experiments show that under the strong noise condition of SNR = −1 dB, the diagnostic accuracy rate of the model reaches 87.47%, which is more than 12% higher than that of comparison models such as 1D-DCTN. Even in an extreme noise environment with SNR = −9 dB, the accuracy rate remains at 90.85%, and 90% of the error samples are concentrated in similar working conditions. There are fewer than eight cross-category misjudgments, fully verifying its reliable adaptability in multi-source interference scenarios of pumping stations.
(2) It has excellent adaptability to small samples and lowers the threshold for labeling costs. To address the issue of scarce labeled data in actual operation and maintenance, the model achieves core feature mining with a small number of samples through tSSA global optimization and Informer information distillation mechanisms. When the labeled sample size is reduced to 10%, its accuracy rate still reaches 61.32%, which is nearly 26 percentage points higher than that of the suboptimal DWCDA-CNN. It is much higher than traditional models such as Fisher-SSA and 1D-DCTN, significantly reducing the reliance on large-scale labeled data and adapting to scenarios where the data accumulation of new monitoring stations is insufficient.
(3) The diagnostic accuracy and optimization efficiency are synergistically optimized, and the performance is comprehensively leading. The efficient optimization of Informer hyperparameters by tSSA has achieved a dual improvement in accuracy and efficiency: in a noise-free environment, the average diagnostic accuracy of the model reaches 98.73%, with the highest accuracy reaching 99.36%, which is 5.17% higher than that of SSA-Informer. Hyperparameter optimization only takes 10 min and 44 s, which is 40% faster than the genetic algorithm and has better search accuracy than PSO-Informer. The error rate is reduced by 12% under similar parameter configurations. Both the confusion matrix and t-SNE visualization show that the model has clear feature clustering for the 12 types of faults, with closely clustered samples of the same type and high boundary discrimination.

## Figures and Tables

**Table 1:** Bearing fault data.

Fault Type	Rotational Speed	Number of Samples	Degree of Failure	Category
Parallel misalignment (C)	1000	400	0.07	0
	1500	400	0.14	1
	2000	400	0.21	2
Rotor–stator friction fault (PM)	1000	400	0.07	3
	2000	400	0.14	4
	2500	400	0.21	5
	3000	400	0.28	6
Misalignment—rubbing coupling fault (PMC)	1000	400	0.07	7
	1500	400	0.14	8
	2000	400	0.21	9
	2500	400	0.28	10
	3000	400	0.35	11

**Table 2:** Characteristic contribution rate.

Dimension Numbering	Cumulative Contribution Rate	Dimension Numbering	Cumulative Contribution Rate
1	0.1384	13	0.8070
2	0.2621	14	0.8289
3	0.3767	15	0.8503
4	0.4841	16	0.8706
5	0.5352	17	0.8904
6	0.5795	18	0.9076
7	0.6220	19	0.9246
8	0.6633	20	0.9410
9	0.7026	21	0.9568
10	0.7308	22	0.9722
11	0.7573	23	0.9868
12	0.7826	24	1.0000

**Table 3:** Hyperparameter optimization results.

Model		Parameter	Value	Time Consumed
tSSA–Informer	*Core architecture parameters*	*e_layers*	3	10 min 44 s
		*d_layers*	1	
		*n_head*	4	
		*distillation* *layers*	2	
	*Hyperparameter*	*d_model*	192	
		*d_ff*	155	
	*Activation function*	*Feedforward network activation function*	*RELU*	
		*Output layer activation function*	*S* *oftmax*	
SSA–Informer	*Core architecture parameters*	*e_layers*	2	11 min 38 s
		*d_layers*	1	
		*n_head*	4	
		*distillation* *layers*	1	
	*Hyperparameter*	*d_model*	176	
		*d_ff*	137	
	*Activation function*	*Feedforward network activation function*	*RELU*	
		*Output layer activation function*	*S* *oftmax*	
GA–Informer	*Core architecture parameters*	*e_layers*	4	18 min 53 s
		*d_layers*	1	
		*n_head*	5	
		*distillation* *layers*	2	
	*Hyperparameter*	*d_model*	217	
		*d_ff*	143	
	*Activation function*	*Feedforward network activation function*	*RELU*	
		*Output layer activation function*	*S* *oftmax*	
PSO–Informer	*Core architecture parameters*	*e_layers*	3	10 min 17 s
		*d_layers*	1	
		*n_head*	4	
		*distillation* *layers*	2	
	*Hyperparameter*	*d_model*	194	
		*d_ff*	171	
	*Activation function*	*Feedforward network activation function*	*RELU*	
		*Output layer activation function*	*S* *oftmax*	

**Table 4:** Model training resource consumption and performance indicators.

Model		tSSA-Informer	SSA-Informer	GA-Informer	PSO-Informer
Training resource consumption	Total time consumption (h)	2.8	4.2	3.6	3.1
	Peak GPU memory (GB)	10.2	13.7	12.5	11.8
	Peak CPU memory (GB)	28.5	32.8	30.1	29.7
Inference performance indicators	GPU side latency (ms)	9.6	13.2	11.8	10.3
	Edge CPU latency (ms)	42.3	58.7	51.2	45.6
	Accelerate module end delay (ms)	18.7	25.4	22.6	20.1
	Inference GPU memory usage (MB)	850	1050	920	880
	Inference CPU memory usage (MB)	480	620	550	510

**Table 5:** Comparison results of algorithm models.

Model	Average Diagnostic Accuracy %	Max Diagnostic Accuracy %
tSSA-Informer	98.73	99.36
SSA-Informer	93.56	94.35
GA-Informer	96.34	97.51
PSO-Informer	97.36	97.69
Informer	91.75	92.92

**Table 6:** The results of the statistical significance test for model performance.

Combination of Comparative Models	tSSA—Average Accuracy Rate of Informer (%)	Average Accuracy Rate of the Comparison Model (%)	Standard Deviation	Paired *t*-Test *p* Value	95% Confidence Interval (Difference in Accuracy Rate, %)
tSSA-Informer vs. SSA-Informer	98.73	93.56	0.42	<0.001	[4.72, 5.62]
tSSA-Informer vs. GA-Informer	98.73	96.34	0.38	<0.001	[2.05, 2.73]
tSSA-Informer vs. PSO-Informer	98.73	97.36	0.35	0.002	[1.01, 1.73]

**Table 7:** Comparison of multiple indicators of each model under different noise intensities.

Model	SNR	Accuracy Rate	Macro-P	Macro-R	Macro-F1
Fisher-SSA	None	98.46	98.23	98.35	98.29
	9 dB	97.57	97.31	97.42	97.36
	5 dB	95.60	95.34	95.47	95.40
	1 dB	93.44	93.18	93.30	93.24
	−1 dB	90.56	90.29	90.41	90.35
	−5 dB	87.63	87.35	87.48	87.41
	−9 dB	84.54	84.26	84.39	84.32
DWCDA-CNN	None	97.85	97.61	97.72	97.66
	9 dB	96.94	96.68	96.80	96.74
	5 dB	94.87	94.60	94.73	94.66
	1 dB	93.08	92.81	92.94	92.87
	−1 dB	90.83	90.55	90.68	90.61
	−5 dB	87.25	86.97	87.10	87.03
	−9 dB	84.81	84.52	84.65	84.58
CEEMD-VMD	None	96.52	96.27	96.39	96.33
	9 dB	95.81	95.55	95.68	95.61
	5 dB	94.13	93.86	93.99	93.92
	1 dB	92.83	92.55	92.68	92.61
	−1 dB	90.12	89.84	89.97	89.90
	−5 dB	86.54	86.25	86.38	86.31
	−9 dB	84.53	84.24	84.37	84.30
1 D-DCTN	None	99.17	98.92	99.05	98.98
	9 dB	98.42	98.16	98.29	98.22
	5 dB	96.74	96.47	96.60	96.53
	1 dB	95.15	94.88	95.01	94.94
	−1 dB	92.16	91.87	92.00	91.93
	−5 dB	88.26	87.96	88.09	88.02
	−9 dB	86.20	85.90	86.03	86.01

**Table 8:** Comparison results of algorithm models with different labeled samples.

Model	Accuracy With Different Sample Sizes of Labels			
	70%	50%	30%	10%
Fisher-SSA	90.37	73.54	46.54	21.62
DWCDA-CNN	91.83	77.19	52.73	35.41
CEEMD-VMD	90.11	75.38	49.72	32.54
1D-DCTN	93.42	79.68	45.42	18.29
tSSA-Informer	93.17	87.25	73.84	61.32

**Table 9:** Comparison of ablation experiment results (%).

Model Variant	Full Sample Scenarios (Acc/Relative Improvement)	Small Sample Scenario (10% Marking, Acc/Relative Improvement)	Strong Noise Scene (SNR = −9 dB, Acc/Relative Improvement)
Informer	91.75/0.0	61.32/0.0	75.40/0.0
Informer + trend cycle separation	94.20/+2.45	67.30/+5.80	81.60/+6.20
Informer + trend cycle separation + sparse attention	96.53/+4.78	74.40/+13.30	85.35/+9.95
tSSA-Informer	98.73/+6.98	61.32/+24.50	90.85/+15.45

## Data Availability

Restrictions apply to the availability of these data. The data were obtained from a third party. The data are not publicly available due to privacy restrictions.
